# Structural basis for Ca^2+^ activation of the heteromeric PKD1L3/PKD2L1 channel

**DOI:** 10.1038/s41467-021-25216-z

**Published:** 2021-08-11

**Authors:** Qiang Su, Mengying Chen, Yan Wang, Bin Li, Dan Jing, Xiechao Zhan, Yong Yu, Yigong Shi

**Affiliations:** 1 Key Laboratory of Structural Biology of Zhejiang Province, School of Life Sciences, Westlake University, Hangzhou, Zhejiang Province China; 2 School of Life Sciences, Institute of Biology, Westlake Institute for Advanced Study, Westlake University, Hangzhou, Zhejiang Province China; 3 Beijing Advanced Innovation Center for Structural Biology, Tsinghua-Peking Joint Center for Life Sciences, School of Life Sciences, School of Medicine, Tsinghua University, Beijing, China; 4 Department of Biological Sciences, St. John’s University, Queens, NY USA

**Keywords:** Transient receptor potential channels, Cryoelectron microscopy

## Abstract

The heteromeric complex between PKD1L3, a member of the polycystic kidney disease (PKD) protein family, and PKD2L1, also known as TRPP2 or TRPP3, has been a prototype for mechanistic characterization of heterotetrametric TRP-like channels. Here we show that a truncated PKD1L3/PKD2L1 complex with the C-terminal TRP-fold fragment of PKD1L3 retains both Ca^2+^ and acid-induced channel activities. Cryo-EM structures of this core heterocomplex with or without supplemented Ca^2+^ were determined at resolutions of 3.1 Å and 3.4 Å, respectively. The heterotetramer, with a pseudo-symmetric TRP architecture of 1:3 stoichiometry, has an asymmetric selectivity filter (SF) guarded by Lys2069 from PKD1L3 and Asp523 from the three PKD2L1 subunits. Ca^2+^-entrance to the SF vestibule is accompanied by a swing motion of Lys2069 on PKD1L3. The S6 of PKD1L3 is pushed inward by the S4-S5 linker of the nearby PKD2L1 (PKD2L1-III), resulting in an elongated intracellular gate which seals the pore domain. Comparison of the apo and Ca^2+^-loaded complexes unveils an unprecedented Ca^2+^ binding site in the extracellular cleft of the voltage-sensing domain (VSD) of PKD2L1-III, but not the other three VSDs. Structure-guided mutagenic studies support this unconventional site to be responsible for Ca^2+^-induced channel activation through an allosteric mechanism.

## Abstract (web summary)

Hetero-oligomeric TRP-like channels such as PKD1L3/PKD2L1 play crucial roles in a range of physiological and pathophysiological processes. Here, the authors present the cryo-EM structures of a minimal functional murine PKD1L3/PKD2L1 complex in the absence and presence of calcium and further supported through structure-guided mutagenic studies, they discuss the mechanism of calcium-induced channel activation.

## Introduction

The polycystic kidney disease (PKD) protein family is named after the founding member, PKD1, which was originally identified in linkage studies for autosomal dominant polycystic kidney disease (ADPKD), one of the most common human genetic diseases^1–4^. The PKD family is further classified into the PKD1 and PKD2 subfamilies based on sequence homology.

The PKD1 subfamily consists of PKD1, PKD1L1, PKD1L2, PKD1L3, and PKDREJ. Each PKD1 member comprises a large N-terminal extracellular domain (ECD) of 1000–3000 residues, 11 transmembrane segments, and a short intracellular C-terminal tail (CTT). The soluble domains in ECD and CTT vary among different members (Supplementary Fig. 1). The PKD2 subfamily, which comprises three members, PKD2, PKD2L1, and PKD2L2, also belongs to the transient receptor potential polycystin (TRPP) family^4–7^. PKD2 proteins can either homotetramerize^8^ or assemble with PKD1 proteins, exemplified by the PKD1/PKD2 and PKD1L3/PKD2L1 complexes^9–12^.

PKD complexes are hypothesized to function as heteromeric TRP-like channels, which play important roles in many physiological processes, such as controlling ciliary Ca^2+^ concentration and establishing the embryonic left–right axis^10,13–17^. Among all PKD heterochannels, PKD1L3/PKD2L1 channel is the only one whose gating properties have been characterized, thus representing a good model for structure-function relationship investigation.

PKD1L3 is expressed to a high level in the liver and testis, but its function in these tissues remains elusive^18^. The widely distributed PKD2L1, on the other hand, is involved in a variety of biological processes, including regulation of neuronal excitability, mechanoreception in cerebrospinal fluid-contacting neurons, as well as calcium homeostasis and Sonic Hedgehog signaling in the primary cilia^13,19–22^. The heterotetrameric PKD1L3/2L1 was found in a subset of taste receptor cells that are responsible for acid sensing and suggested to be a sour taste receptor candidate^10,19,23–25^. However, this hypothesis has been recently challenged by animal studies and the identification of the Otop1 proton channel as the primary sour taste receptor^26–29^. The real role of the PKD1L3/2L1 complex in sour taste needs further investigation.

The PKD1L3/2L1 complex forms a calcium-permeable, nonselective cation channel, with the permeability preference of P_Ca_:P_Na_:P_Mg_ at ~11:1:0.3^10,12,30,31^. The recombinantly expressed complex can be activated upon washout of the applied acid solution^10,19,24,30^. It can also be gated by transient extracellular calcium exposure followed by inactivation in an intracellular Ca^2+^-dependent manner^32,33^. Despite rigorous electrophysiological characterizations, the molecular basis for stimuli sensing remains elusive, necessitating high-resolution structural elucidation.

The only available structure of the polycystin heterocomplexes between PKD and TRPP proteins, or the heterotetrameric TRP-like channel, is that of PKD1/2^34^. However, the putative channel activity of this complex remains controversial^15,16,35,36^, preventing structure-guided mechanistic interpretation. We, therefore, have focused on PKD1L3/2L1 for structural analysis with the aim of establishing a prototype for structure–function relationship investigation of the hetero-oligomeric TRP-like channels.

Here, we report the cryo-EM structures of the PKD1L3/2L1 complex before and after the addition of 20 mM Ca^2+^ at resolutions of 3.4 Å and 3.1 Å, respectively. These structures reveal a different selectivity filter (SF) and pore domain (PD) from that of the PKD1/2 heterocomplex. Structural comparison and structure-guided mutagenic analyses elucidate an unconventional Ca^2+^ binding site on the extracellular side of the VSD of only one PKD2L1 subunit that may be responsible for Ca^2+^-induced channel activation.

## Results

### The overall structure and function of PKD1L3-CTD/PKD2L1

**Fig. 1:**
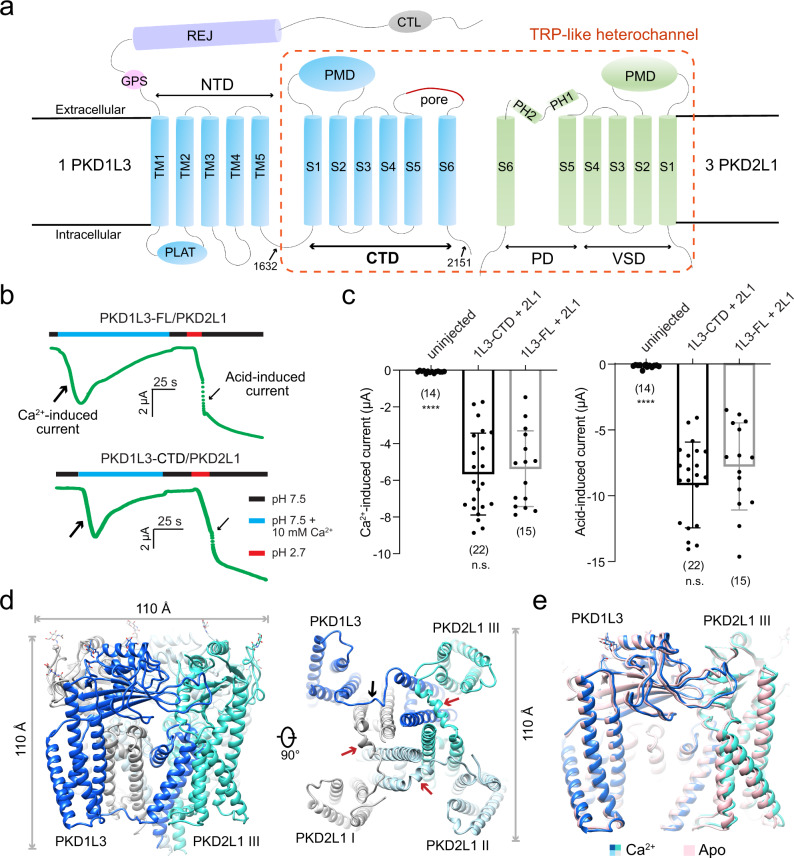
The PKD1L3-CTD/PKD2L1 heterocomplex retains Ca^2+^ and acid-induced channel activity. **a** Topology diagram of PKD1L3 and PKD2L1. PKD1L3, and PKD2L1 proteins assemble to a heterocomplex with a stoichiometric ratio of 1:3. The constructs used for structural determination in this study, mouse PKD1L3-CTD (residues 1632–2151) and PKD2L1 (residues 64–629), are indicated by the dashed box. CTL C-type lectin domain, REJ sperm receptor for egg jelly, PLAT Polycystin-1, Lipoxygenase, α-toxin domain, PMD polycystin-mucolipin domain, PD pore domain, VSD voltage-sensing domain. **b** The Ca^2+^ and acid-induced channel activity of the PKD1L3-CTD/PKD2L1 heterocomplex is similar to that of the full-length (FL) channel. The Ca^2+^ and acid-induced currents were recorded from *Xenopus* oocytes expressing either full-length PKD1L3 or PKD1L3-CTD with PKD2L1. Shown here are representative traces for gap-free recording at −80 mV. **c** Scatter plots and bar graphs of the Ca^2+^-induced currents at −80 mV recorded from oocytes expressing the indicated proteins. The number of oocytes is shown below each bar. Data are presented as mean ± SD in the bar graph. Currents in each group are compared with that of PKD1L3-FL/2L1-injected group with two-sided Student’s *t* test. n.s.: not significant; ****P < 0.0001. **d** The side (left) and intracellular (right) views of the cryo-EM structure of the PKD1L3-CTD/PKD2L1 complex. For simplicity, we will call it PKD1L3/2L1. The glycosyl moieties are shown as sticks. The black and red arrows highlight the different structures of the S4–S5 linkers in PKD1L3 and the three PKD2L1 subunits, respectively. **e** Structural comparison of PKD1L3/2L1 with and without added Ca^2+^. The two structures will be referred to as “Ca^2+^-loaded” and “apo”. All structural figures were prepared in UCSF chimera^65^ if not otherwise indicated.

Among the 11-transmembrane (TM) helices of PKD1L3, TM1-TM5 constitute the amino-terminal domain (NTD) and TM6-TM11 (S1-S6) form the carboxy-terminal domain (CTD), also known as the TRP-like domain (Fig. 1a)^34,37^. The PKD1L3-CTD comprises the voltage-sensing domain (VSD), the polycystin-mucolipin domain (PMD^38,39^, also known as the polycystin domain^40,41^ or TOP domain^42^), and the PD.

To investigate whether the PKD1L3-CTD/PKD2L1 preserves an intact channel function, we expressed the full-length (FL) PKD1L3/PKD2L1 and truncated PKD1L3-CTD/PKD2L1 complexes in *Xenopus laevis* and tested their function by applying Ca^2+^ and acid stimuli, respectively. The results show that, similar to that of the FL PKD1L3/PKD2L1 complex, obvious Ca^2+^ and acid-induced currents were recorded from the PKD1L3-CTD/2L1 complex, suggesting the presence of Ca^2+^ and pH sensor on this minimal complex (Fig. 1b, c and Supplementary Fig. 2a). Our results are consistent with a previous report showing the complex formed by the PKD1-CTD and PKD2 resembles the channel function of the full-length complex^37^. Being curious about how this small-channel core plays a full function, we focused on the truncated complex for cryo-EM analysis. For simplicity, it will still be referred to as PKD1L3/2L1.

To obtain a good cryo-EM sample, we employed the double-blots method to increase the accessibility of protein particles into vitrified ice, and added 0.2% fluorinated Fos-Choline-8 to relieve the preferred orientation of the particles. In order to solve the problem of the incorrect three-dimensional (3D) reconstruction due to the fourfold pseudosymmetry, we collected about 10,000 movie stacks and selected a large number of particles for the 3D classification and refinement. Details of protein purification, sample preparation, and cryo-EM analysis can be found in “Methods”. After overcoming these technical hurdles, we obtained the 3D reconstructions of PKD1L3/2L1 at 3.1 and 3.4 Å resolutions in the presence and absence of 20 mM CaCl_2_, respectively (Supplementary Figs. 2, 3 and Supplementary Table 1).

The excellent EM maps supported reliable model building for most of the transmembrane and extracellular segments (Supplementary Fig. 3). Consistent with a previous photo bleaching study and similar to the structure of PKD1/2, the PKD1L3/2L1 complex adopts a 1:3 stoichiometric assembly with a conventional domain-swapped voltage-gated ion channels (VGICs) architecture^12,34^. To facilitate illustration, we will describe the three PKD2L1 subunits as I, II, and III following an anticlockwise order in the intracellular view, with VSD_III_ contacting the PD of PKD1L3 (Fig. 1d, right).

The three-dimensional structure of the PKD1L3/2L1 complex nearly resembles a cube with a side length of ~110 Å (Fig. 1d). The S4–S5 linkers, which are invisible in the structure of PKD2L1 homotetramer^41,43^, were resolved in all three PKD2L1 subunits in the heterocomplex (Fig. 1d, right). Despite similar architecture between PKD1L3 and PKD2L1 in the VSDs, PMDs, and PD, major structural distinction occurs in the S4–S5 linker, which, similar to most VGICs, is a helix in PKD2L1, but exists as an extended loop in PKD1L3 (Fig. 1d, indicated by the black arrow). In the presence of Ca^2+^, the overall conformation remains largely unchanged, except for minor but critical local shifts that will be depicted in a later session (Fig. 1e).

### A closed PD constricted by unique S4–S5 linkers

**Fig. 2:**
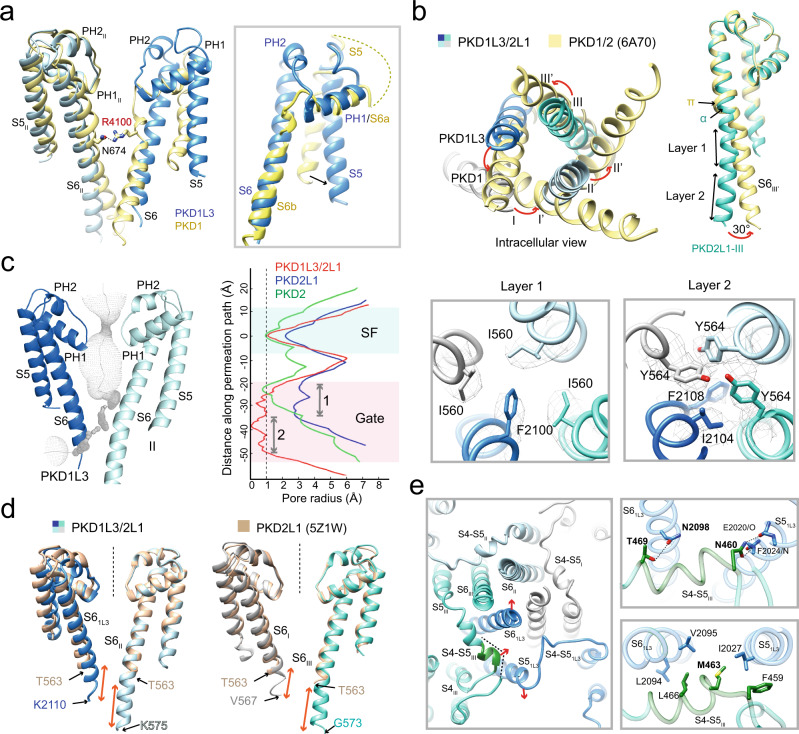
A closed pore with an elongated intracellular gate. **a** Difference in the architecture of PD between PKD1L3/2L1 and PKD1/2 (PDB code: 6A70). PKD1L3 has a conventional PH1-SF-PH2 segment that is missing in PKD1^34^. Inset: Comparison of the PD segments of PKD1 (yellow) and PKD1L3 (blue). The S6 segment of PKD1 bends in the middle, resulting in a S6a half helix that aligns with a typical pore helix PH1. The sequence connecting S6a and S5 is invisible in PKD1. **b** Conformational shifts between the S6 tetrahelical bundles of PKD1L3/2L1 and PKD1/2. The PD of apo PKD1L3/2L1 is superimposed with that of PKD1/2. A secondary structural element transition in the middle of S6 from an α helical turn in PKD2L1 to a π helix in PKD2 results in an iris-like rotation of the S6 tetrahelical bundle. The conformational shifts of the corresponding segments from PKD1L3/2L1 to PKD1/2 are indicated by red arrows. **c** The PD is sealed by an elongated intracellular gate. The permeation path of the apo heterotetramer, calculated by HOLE^69^, is illustrated by gray dots. The pore radii of PKD1L3/2L1 (red), homotetrameric PKD2L1 (blue, PDB code: 5Z1W), and homotetrameric PKD2 (green, PDB code: 5T4D) are compared (right). The intracellular gate of PKD1L3/2L1 is extraordinarily elongated and will be illustrated as two layers. Right two panels: Composition of the two layers of the intracellular gate. Shown here are extracellular views. The densities are contoured at 5 σ. **d** PD comparison between PKD1L3/2L1 and PKD2L1 homotetramer. Longer S6 segments of PKD2L1 were resolved in PKD1L3/2L1 than those in the PKD2L1 homotetramer. Note that the PKD2L1 subunits exhibit nearly identical conformations in the two channels. **e** Structural deviations of the S4–S5 linkers of the four subunits in PKD1L3/2L1. Left: Whereas the linkers between S4 and S5 in PKD2L1, similar to those in other VGIC proteins, forms a short helix, that in PKD1L3 is a loop. Even among the three PKD2L1 subunits, the S4–S5 linker in VSD_III_ is distinct from the other two. The C-terminal short helix (dark green) of the S4–S5_III_ bends toward the PD, pushing S6 of PKD1L3 inward. Right: The S4–S5 helix of PKD2L1-III interacts with the S5 and S6 helices of PKD1L3 through specific hydrogen bonds (upper panel) and extensive van der Waals contacts (lower panel). The hydrogen bonds are indicated by black dashed lines.

The S5 and S6 helices and their intervening segments, pore helices PH1 and PH2, from the four subunits of PKD1L3/2L1 constitute the PD (Fig. 2a). Drastic structural differences are observed between the PD of the two heterocomplexes, PKD1L3/2L1 and PKD1/2. The extracellular half of PKD1-S6 bends in the middle to result in two half helices, S6a and S6b, with S6a tilted to occupy the position of a typical pore helix PH1 in other VGIC channels. In contrast, the PD segments of PKD1L3 conforms to a classic VGIC fold (Fig. 2a).

In addition, PKD2L1 displays a different conformation from PKD2 in these two heterocomplexes. There is an α → π transition in the middle of the S6 segment from PKD2L1 to PKD2. Consequently, the overall S6 tetrahelical bundle undergoes an iris-like rotation, resulting in distinct intracellular constriction sites along the permeation path in the two heterocomplexes (Fig. 2b, c). The pore radius at the narrowest point along the permeation path of PKD1L3/2L1 is <1 Å, representing a closed state (Fig. 2c).

It is noted that the S6 helices were resolved one to three helical turns longer in the heterotetramer than those in the homotetrameric PKD2L1^34,40^ (Fig. 2d), allowing for the resolution of an exceptionally lengthy intracellular gate. To facilitate analysis, the gate will be described as two layers (Fig. 2c). Layer 1 is formed by Phe2100 in PKD1L3 and three Ile560 in the PKD2L1 subunits, and Layer 2 is sealed by nonpolar residues, Ile2104 and Phe2108 in PKD1L3, and Tyr564 in PKD2L1 (Fig. 2c, two panels on the right). Interestingly, the overall conformations of the S6 helices of the three PKD2L1 subunits in the heterotetramer remain nearly identical to those in the open homotetrameric PKD2L1 (Fig. 2d). Therefore, the closure of Layer 1 is mainly achieved by insertion of PKD1L3-Phe2100 into the center of the three Ile560 residues on PKD2L1 (Fig. 2c). However, the single or double mutations, F2100A, F2108A, and F2100A/F2108A of PKD1L3, whose aromatic side chains are removed, are not enough to open the gate and result in constitutively active channels (Supplementary Fig. 4).

The unique conformation of PKD1L3-S6 appears to be stabilized by the S4–S5 linker in the adjacent PKD2L1-III (Fig. 2e). As shown in Fig. 1d, the VSD and PD, or the S4 and S5 segments, of PKD1L3 is connected by an extended loop. A close examination shows that the conformation of this important functional entity also varies among the three PKD2L1 subunits. The S4–S5 linker exists as a loop followed by a short helix in subunits I and II. However, in PKD2L1-III, the loop-corresponding segment folds to a short helix that bulges into the interface between the S5 and S6 segments of PKD1L3, pushing S6 toward the S6 helices in the PKD2L1 subunits to seal the intracellular gate (Fig. 2e, left). The interaction between the S4–S5 linker of PKD2L1-III and the S5 and S6 segments of PKD1L3 is mediated by both hydrogen bonds and van der Waals contacts (Fig. 2e, right).

The unprecedented conformation of the intracellular gate and the S4–S5 linkers of PKD1L3/2L1 exemplifies the diversity of hetero-oligomeric TRP-like channels, necessitating structural determination of specific heterochannels to understand their unique gating and permeation mechanisms.

### A Lys switch in the asymmetric SF

**Fig. 3:**
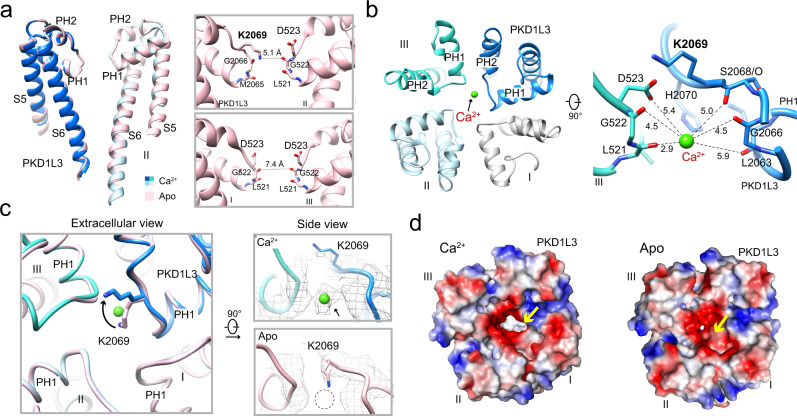
A Lys switch for Ca^2+^ entrance to the “KDDD” selectivity filter (SF). **a** An asymmetric SF. Left: The pore domain of the apo and Ca^2+^-loaded complexes exhibits nearly identical overall conformation. Right: The distinct composition of the SF segments in PKD1L3 and PKD2L1 results in an asymmetric SF in the heterotetramer. Shown in the insets are the side views of the SF in the two diagonal subunits of the apo structure. **b** A Ca^2+^ ion is found in the SF in the Ca^2+^-loaded structure. Left: Ca^2+^ is positioned off the central axis and closer to PKD1L3. Shown here is an extracellular view of the PD. Right: The bound Ca^2+^ ion is mainly coordinated by three carbonyl oxygen groups on the SF loop of PKD1L3. The carboxylate group of Asp523 and two carbonyl oxygens from the two preceding residues in PKD2L3-III may also contribute to water-mediated coordination. The distances are indicated as black dashed lines and labeled in unit Å. **c** The upper site of the SF is open for Ca^2+^ entry with an outward swing of the long side chain of Lys2069. Left: Distinct conformations of Lys2069 between the apo (pink) and the Ca^2+^-loaded (domain colored) structures. Right: The Ca^2+^ was clearly resolved in the map of Ca^2+^-loaded complex. The densities, shown as gray mesh, are contoured at 4 σ. **d** An enlarged outer mouth and redistributed surface charges of the SF upon addition of Ca^2+^. Shown here are extracellular views of the electrostatic surface potential of the PD calculated in PyMol^70^.

The overall PD structures in the apo and Ca^2+^-loaded PKD1L3/2L1 complexes are nearly identical (Fig. 3a, left). Because of the sequence and conformational variations between PKD1L3 and the three PKD2L1 subunits, the SF is asymmetric. The entrance to the SF vestibule is guarded by “KDDD”—Lys2069 from PKD1L3 and Asp523 from the three PKD2L1 subunits. An inner site is constituted by the carbonyl oxygen (C = O) groups of Gly2066 and Met2065 in PKD1L3 and Gly522 and Leu521 in PKD2L1 (Fig. 3a, right).

The well-resolved local densities reveal that the constriction site in the SF is determined by PKD1L3-Lys2069. The distance between its amine group and the C = O of Gly522 from the opposing PKD2L1-II is 5.1 Å, corresponding to a van der Waals radius of ~1 Å (Fig. 3a, right). Structural comparison between PKD1L3/2L1 and the PKD2L1 homotetramer, which has a conductive SF, suggests that replacement of PKD2L1-Asp523 with PKD1L3-Lys2069 not only narrows the SF path, but also reduces the electronegativity that is important for cation attraction (Supplementary Fig. 5a, b). Therefore, Lys2069 appears to block the SF of the apo complex in a non-conductive conformation.

The “KDDD” motif of PKD1L3/2L1 is reminiscent of the structures of eukaryotic Na_v_ channels and the recently published NALCN, whose SFs are featured with “DEKA” and “EEKE”, respectively^44–46^ (Supplementary Fig. 5a, right). In both PKD1L3/2L1 and NALCN, the side chain of Lys neutralizes the carboxylate from the Asp or Glu. The presence of a positively charged amine group may represent a stronger resistance to divalent cations, providing a putative explanation for the higher selectivity of Na^+^ than Mg^2+^ (Supplementary Fig. 5b, right). Supporting this analysis, K2069D mutation of PKD1L3 increased the permeability ratio of P_Mg_/P_Na_ by approximal sixfolds^12^.

The half-maps of reconstructions of the apo and Ca^2+^-loaded complex revealed an unambiguous density that was present only in the SF vestibule of the Ca^2+^-loaded complex, but not the apo one (Supplementary Fig. 5c). A Ca^2+^ ion was thereby assigned to this density. The ion is shielded by the SF loop of PKD1L3 and PKD2L1-III, deviating from the central axis of the pore (Fig. 3b, left). The Ca^2+^ is coordinated by the C = O groups of Leu2063, Gly2066, and Ser2068 in PKD1L3, and by the C = O of Leu521 and Gly522 as well as the carboxylate of Asp523 in PKD2L1-III (Fig. 3b, right). The distance between the C = O groups and the Ca^2+^ ion ranges between 2.9 and 5.9 Å, indicating a partially hydrated state of the bound ion^47^.

Comparison of the apo and Ca^2+^-loaded structures reveals an interesting local switch within the SF vestibule. In the apo structure, the side chain of Lys2069 from PKD1L3 projects into the central cavity and occupies the abovementioned Ca^2+^ binding pocket (Fig. 3c). Upon addition of Ca^2+^, Lys2069 swings upwards, resulting in an enlarged outer mouth with altered electrostatic potential distribution (Fig. 3d). The switch-like motion of a Lys residue in the SF has not been observed in eukaryotic Na_v_ channels or NALCN, in which the conserved Lys exhibits identical conformation under all conditions^45,46,48–52^. It is noted that the apo structure was attained in the presence of 150 mM Na^+^, suggesting that the switch is induced by Ca^2+^, but not Na^+^.

### An unconventional Ca^2+^-binding site in VSD_III_

**Fig. 4:**
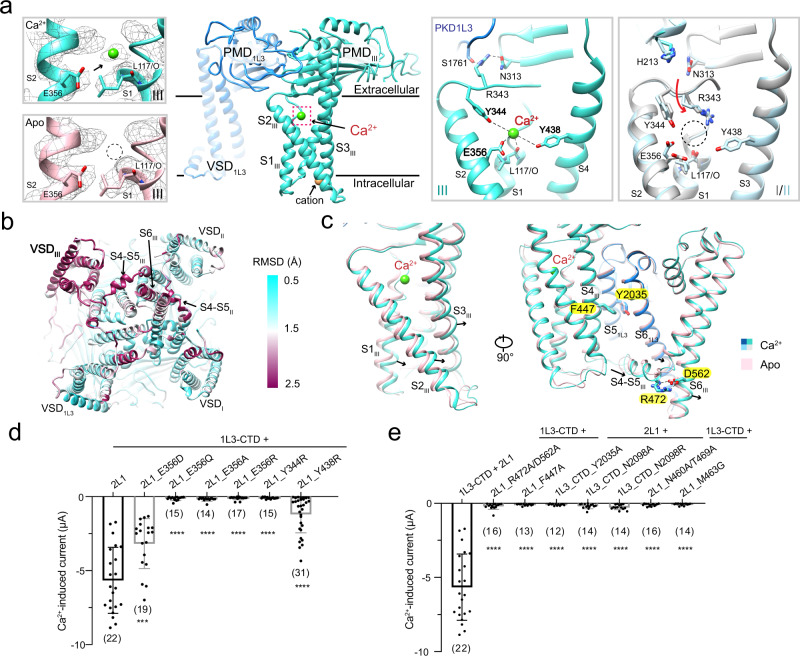
An unconventional Ca^2+^ binding site in VSD_III_ of the heterocomplex may be responsible for Ca^2+^ activation. **a** A Ca^2+^ ion is found in the extracellular cleft of VSD_III_. Left two panels: An extra density is found in VSD_III_ only in the presence of added Ca^2+^. The densities are contoured at 4 σ. Right two panels: Different local conformations of the three VSDs of PKD2L1 reveal the molecular basis for the VSD_III_-only binding site for Ca^2+^. Arg343 in VSD_III_ projects toward the PMD domain to interact with Asn313 in PMD_III_ and Ser1761 in PKD1L3, whereas in VSD_I_ (silver) and VSD_II_ (pale cyan) it points to the interior of the VSD and occupies the Ca^2+^-binding site. The down conformation of Arg343 in these two VSDs is likely owing to repulsion by His213 from the neighboring PKD2L1 subunit. **b** VSD_III_ deviates to a larger degree between the apo and Ca^2+^-loaded structures than the other three VSDs. Shown here is the heatmap of RMSD between the two structures. **c** Relatively minor but important conformational changes of VSD_III_, S4–S5_III_, and PD between apo and Ca^2+^-loaded states implicate the molecular basis for Ca^2+^-induced channel activation. Left: Shifts of the VSD_III_ segments. Right: Key residues that may mediate channel activation upon Ca^2+^ binding to VSD_III_. **d** Functional validation of the residues in panel **a** that constitute the Ca^2+^-activation site. **e** Functional validation of the residues in panel **c** that may be involved in the conformational coupling for Ca^2+^-induced channel activation. Data are presented as mean ± SD in the bar graph. Currents of other groups are compared with that injected with PKD1L3-CTD/PKD2L1 with two-sided Student’s *t* test. ***P < 0.001; ****P < 0.0001. No significant or mild changes in acid-induced current were observed in these mutants (Supplementary Fig. 10).

Apart from the Ca^2+^-binding site within the SF, additional ion-binding sites are observed in the VSDs of PKD2L1 subunits (Fig. 4a). All four VSDs are well-resolved in both PKD1L3/2L1 reconstructions (Supplementary Fig. 3). In the half-maps of both apo and Ca^2+^-loaded complexes, a small density contiguous with that of Asn387 on S3 is seen in all three VSDs of PKD2L1 near the intracellular side (Supplementary Fig. 6a). A similar ion-binding site has been observed in several TRP structures and suggested for ligand gating in TRPC5 and Ca^2+^ potentiation in TRPA1^53–55^. However, because the density is observed in both apo and Ca^2+^-loaded PKD1L3/2L1, we remain cautious about its identity and will describe it as a cation, as the binding site is constituted by acidic or polar residues (Supplementary Fig. 6b). This site is missing in the VSD of PKD1L3 owing to sequence variations (Supplementary Fig. 6c, d).

Scrutiny of the maps of the apo and Ca^2+^-loaded complexes reveals an unconventional Ca^2+^-binding site in the VSD of PKD2L1-III only. A spherical density is found in the extracellular cleft of VSD_III_ only in the Ca^2+^-loaded complex, but missing in other VSDs or in any of the VSDs in the apo complex (Fig. 4a, left and Supplementary Fig. 7a). Therefore, a Ca^2+^ ion was assigned to this density in the pocket formed by S1_III_, S2_III_, and S3_III_ (Fig. 4a). The Ca^2+^ is predominately coordinated by carboxylate of Glu356. The neighboring hydroxyl groups of Tyr344 and Tyr438 and C = O of Leu117 also contribute to ion coordination (Fig. 4a).

The different local conformations among the three VSDs of PKD2L1 provide a plausible explanation for the lack of Ca^2+^ ion in VSD_I_ and VSD_II_. In VSD_III_, which is adjacent to the PMD and PD of PKD1L3, Arg343 reaches out to interact with Ser1761 and Asn313 at PMD_PKD1L3_ and PMD_III_, respectively. However, Arg343 points into the interior of VSD_I_ and VSD_II_ likely owing to repulsion from His213 in the neighboring PKD2L1 subunit. The positively charged Arg343 thus impedes Ca^2+^ binding (Fig. 4a).

### The unconventional site in VSD_III_ may be responsible for Ca^2+^ activation

To investigate the functional significance of this VSD_III_-unique Ca^2+^-binding site, we carefully analyzed the relatively minor conformational changes of the complex upon Ca^2+^ loading and performed structure-guided mutagenic characterizations accordingly.

Root-mean-square deviation (RMSD) analysis of the apo and Ca^2+^-loaded structures shows that the VSD_III_ exhibits a larger degree of conformational changes than the other three VSDs (Fig. 4b). In the presence of Ca^2+^, VSD_III_ undergoes a downward and lateral motion that pushes S4–S5_III_ toward S5 and S6 of PKD1L3 (PD_1L3_) (Fig. 4c). This motion, although does not directly lead to the opening of the gate in the solved structure, suggests that conformational changes on the extracellular side of VSD_III_ can be transmitted to the intracellular gate of the PD, thereby suggesting a potential mechanism for Ca^2+^-induced channel activation.

To test this hypothesis, we introduced a number of single point mutations to PKD2L1 and PKD1L3 residues that are either involved in Ca^2+^-binding or potentially mediating the transmission of conformational changes (Fig. 4d, e). The interacting pairs, Phe447/Tyr2035 and Arg472/Asp562 at the interface of S4_III_/S5_1L3_ and S4–S5_III_/S6_III_, respectively, may be involved in the conformational coupling (Fig. 4c, right). In addition, the interaction between S4–S5_III_ and S5_1L3_/S6_1L3_, formed by specific hydrogen bonds and extensive van der Waals effects, may also participate in this motion (Fig. 2e).

Supporting a critical role of the unconventional Ca^2+^-binding site, mutations of the Ca^2+^-coordinating residues in VSD_III_, except E356D, abolished or significantly reduced Ca^2+^-induced current (Fig. 4d and Supplementary Fig. 8a). Interestingly, the mutation R343A, also reduces Ca^2+^-induced current (Supplementary Fig. 9). We assume that although Arg343 is not involved in Ca^2+^ binding directly, this mutant may affect the local conformation of the Ca^2+^-binding site in VSD_III_ and thus show a slightly decreased ion current. Consistent with our analysis of the potential conformational coupling route, mutations of the interface residues all completely eliminated the Ca^2+^-induced current (Fig. 4e and Supplementary Fig. 8b). Of particular note, all the mutants retain similar acid-responding currents to the PKD1L3-CTD/2L1 complex, indicating intact protein expression and membrane trafficking (Supplementary Figs. 9c and 10). Meanwhile, it suggests separate determinants for Ca^2+^-dependent and acid-induced activation of the complex.

## Discussion

Despite rapid progress in the structured pursuit of TRP channels since 2013, structural information on the hetero-oligomeric TRP-like channels remains limited. Up to date, PKD1/2 and PKD1L3/2L1 are the only hetero-oligomeric TRP channels whose structures are available. While the channel activity of PKD1/2 remains controversial, our present study identifies a minimal PKD1L3/2L1 complex that retains Ca^2+^ and acid-induced channel activity, hence establishing a model for structure–function relationship studies of hetero-oligomeric TRP-like channels.

**Fig. 5:**
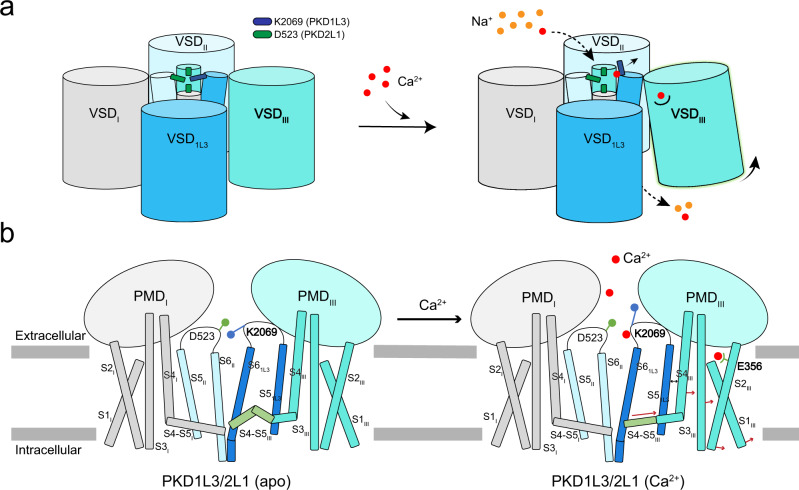
A working model for Ca^2+^ activation of PKD1L3-CTD/2L1. **a** An overall schematic illustration of the conformational changes of the heterochannel upon Ca^2+^ binding to the SF and VSD_III_. The Ca^2+^ and Na^+^ ions are shown as red and orange circles, respectively. **b** A more detailed illustration of the change of channel segments corresponding to the two states shown above. Two diagonal views are shown.

The structures of PKD1L3/2L1 in apo and Ca^2+^-loaded states reveal two Ca^2+^-binding sites at SF and VSD_III_, respectively. While the Lys switch in the SF may respond to Ca^2+^ loading spontaneously, a cascade of conformational changes may lead to pore opening upon Ca^2+^ binding to the extracellular site in VSD_III_ (Figs. 3c, 4, and 5). It is noted that the Ca^2+^-loaded structure remains closed, likely due to the prolonged incubation with Ca^2+^ before plunge freezing. As shown in previous report^32^ and in Fig. 1b, the heterochannel undergoes slow inactivation after Ca^2+^-induced activation. Therefore, the present structure may represent a potentially inactivated state.

The PMD domains is important for channel assembly of the PKD1L3/2L1 complex^56^. We found the binding between PMD_1L3_ and PMD_2L1_ is different from that between PMD_2L1_ and PMD_2L1_, and this seems to be the reason why we still get 3:1 stoichiometry in PKD1L3/PKD2L1 when the C-terminal coiled-coil domain, which has been found to be essential for 3:1 stoichiometry determination^12^, is absent. In this structure, we found that a loop (residues: Lys173 to Gly183) in PMD_2L1-III_ was missing at the interface of PMD_1L3_/PMD_2L1-III_. The loop can interact with adjacent PMD_2L1_ and enhance the assembly between PMD_2L1_ subunits (Supplementary Fig. 11). The missing loop of PMD_2L1-III_ in the current structure indicates that it is flexible and does not bind to PMD_1L3_. Therefore, the binding of PMD_1L3_/PMD_2L1_ should be weaker than that of PMD_2L1_/PMD_2L1_. This observation, combined with our previous biochemical study^56^, made us conclude that the strength of the interaction between PMDs is ranked as follows: PMD_2L1_/PMD_2L1_ >PMD_1L3_/PMD_2L1_ »PMD_1L3_/PMD_1L3_. Therefore, the structure which comprises two or three PMD_1L3_ in the tetrameric complex will be much less stable than the complex that comprises only one PMD_1L3_. Thus, one PMD_1L3_ plus three PMD_2L1_ is preferred in the complex structure. A similar structural feature has been found in the structure of the PKD1/PKD2 complex^34^.

Despite the molecular insight into a potential Ca^2+^-activating mechanism derived from our structural and electrophysiological analyses, the molecular basis for acid activation remains to be elucidated. In addition, the structure of the Ca^2+^-loaded PKD1L3/2L1 complex in an open state is required to fully understand the Ca^2+^-activation mechanism. Our structural and functional analyses reported here establish the framework for further mechanistic dissection of PKD1L3/2L1 as well as other hetero-oligomeric TRP-like channels.

## Methods

### Protein expression and purification

The cDNAs of mouse PKD1L3 (Uniprot: Q2EG98) and mouse PKD2L1 (Uniprot: A2A259) were cloned separately into the pCAG vector with triple FLAG tag and Twin-Strep-tag at N-terminus^57^. The primers used in this study are listed in Supplementary Table 2. For structural analysis, the truncated constructs of mPKD1L3 (residues 1632–2151) and mPKD2L1 (residues 64–629) were used to achieve a higher yield. For expression, HEK293F cells, whose cell density reached 2.0 × 10^6^ cells per mL, were transiently transfected with the expression plasmids and polyethyleneimines (PEIs) (Polysciences). 2 mg plasmids in total (1.5 mg mPKD1L3 plus 1.5 mg mPKD2L1) were premixed with 3 mg PEIs in 45 mL fresh medium for 15–30 min, after which, the mixture was added to 1-L cell culture. Then the transfected cells were cultured under 37 °C supplemented with 5% CO_2_ in a Multitron-Proshaker (Infors, 130 r.p.m.) for 48 h before harvest.

To purify PKD1L3/PKD2L1 complex, the cells were harvested by centrifugation at 3600×*g* for 10 min and resuspended in the lysis buffer (20 mM HEPES, pH 7.5, 150 mM NaCl, 10% (w/v) glycerol, 5 mM EDTA, and Amresco protease inhibitor cocktails (2 μg/ml aprotinin, 2 μg/ml leupeptin, 2 μg/ml pepstatin). The suspension was frozen in liquid nitrogen and temporarily stored at −80 °C for further experiments. For protein purification, the thawed suspension was supplemented with 1 mM PMSF. After homogenization on ice, the membrane fraction was solubilized at 4 °C for 2 h with 2% DDM (Anatrace), 0.5% soybean lipids (Sigma), and 0.4% CHS (Anatrace). After centrifugation at 13,000×*g* for 1 h, the supernatant was collected and applied to anti-Flag M2 affinity resin (MilliporeSigma). The resin was rinsed with the buffer containing 20 mM HEPES, pH 7.5, 150 mM NaCl, 10% (w/v) glycerol, 0.1% (w/v) GDN, and the aforementioned protease inhibitor cocktails. The target protein was eluted with the wash buffer plus 400 μg/ml FLAG peptide. The eluent was then applied to the Strep-Tactin resin (IBA), and the purification protocol was similar to the previous step except that the elution buffer was supplemented by an additional 5 mM d-Desthiobiotin (IBA). The eluate was concentrated using a 50-kDa cutoff Centricon (Millipore) and further purified by size-exclusion chromatography (SEC, Superose^®^ 6 Increase, 10/300 GL, GE Healthcare) in the buffer containing 20 mM HEPES, pH 7.5, 150 mM NaCl, and 0.01% GDN. The peak fractions were collected and concentrated to ~10 mg/ml for cryo-EM analysis. The PKD1L3/PKD2L1 complex in the presence of 20 mM CaCl_2_ was purified following the same procedure, except for the addition of 20 mM CaCl_2_ in the SEC buffer.

### Cryo-EM sample preparation and data collection

Holey carbon grids (Quantifoil, Au, 300-mesh, R1.2/1.3) were glow-discharged in a vacuum for 2 min and mid force for 26 s using Plasma Cleaner PDC-32G-2 (HARRICK PLASMA Company). To avoid preferred orientations, 0.2% (w/v) Fos-Choline-8 (FC8, Anatrace) was added to the protein solution. Aliquots of 3.5 μL freshly purified PKD1L3/PKD2L1 complex were placed on glow-discharged grids. After 3 s of blotting, the grids were plunged into liquid ethane cooled with liquid nitrogen using Vitrobot Mark IV (Thermo Fisher) at 8 °C and 100% humidity.

The prepared grids were subsequently transferred to a Titan Krios electron microscope (Thermo Fisher) operating at 300 kV and equipped with Gatan K2 Summit detector, and GIF Quantum energy filter. A total of 7599 and 9136 zero-loss movie stacks were automatically collected for PKD1L3/PKD2L1 complex alone or supplemented with 20 mM CaCl_2_, respectively, using AutoEMation II with a slit width of 20 eV on the energy filter and a preset defocus range from −1.0 µm to −2.0 µm in super-resolution mode at a nominal magnification of ×81,000^58^. Each micrograph stack, containing 32 frames, was exposed for 5.6 s with a total electron dose of 50 e^−^/Å^2^. The stacks were motion-corrected using MotionCor2 with a binning factor of 2, resulting in a pixel size of 1.087 Å^59^. Meanwhile, dose weighting was performed. The defocus values were estimated with Gctf^60,61^.

### Cryo-EM data processing

The diagram for the data processing of PKD1L3/PKD2L1 complex with or without the addition of 20 mM CaCl_2_ is presented in Supplementary Fig. 2. To overcome incorrect 3D reconstruction, a large body of data was collected. In total, 7599 micrographs for the apo PKD1L3/PKD2L1 complex and 9136 micrographs for the complex in the presence of 20 mM CaCl_2_ were collected. After 2D classification, a total of 1,949,360 (apo) or 2,886,371 (with Ca^2+^) selected particles were subject to global angular search 3D classification with the Class number set to 1 and the step size of 7.5°. The mPKD2L1 map (EMDB code: 6877), low-pass filtered to 10 Å, was used as the initial model. For each of the last five iterations, a local angular search 3D classification was performed with the class number of 8, step size of 3.75°, and local search range of 15°. A total of 1,616,127/910,111 good particles were selected and subjected to further 3D classification, from which 549,716/149,903 particles were selected and subject to 3D auto-refinement, resulting in a final resolution of 3.4 Å for PKD1L3/PKD2L1 complex alone and 3.1 Å for PKD1L3/PKD2L1 complex with 20 mM CaCl_2_. To improve the map quality, the 1,616,127/910,111 particles for the apo and Ca^2+^-treated complexes were processed with a mask during the “skip alignment” 3D classification, yielding maps with improved quality albeit the same nominal resolutions of 3.4 and 3.1 Å, respectively. 2D classification, 3D classification, and auto-refinement were performed with RELION 3.1^62^. The resolution was determined according to the gold-standard Fourier shell correlation 0.143 criteria with a high-resolution noise substitution method^63,64^.

### Model building and refinement

Model building of PKD1L3/PKD2L1 was carried out based on the 3.4 or 3.1 Å reconstruction map, respectively. The structure of mPKD2L1 (PDB code: 5Z1W) was used as the initial model to be docked into the map with Chimera, which was manually adjusted and mutated to the corresponding residues of mPKD1L3 in COOT to yield the final model^41,65,66^. All structural refinements were performed in PHENIX in real space with secondary structure and geometry restraints^67^. Overfitting of the models was monitored by refining the model against one of the two independent half-maps from the gold-standard refinement approach and testing the refined model against the other map^68^.

### Western blot of oocyte lysates

Oocytes were collected into Eppendorf tubes. Lysis buffer (10 µl/oocyte), which contains 1× PBS, 1% n-dodecyl-β-d-Maltoside (DDM), protease inhibitor mixture (Sigma-Aldrich), 1 mM EDTA, and 10% glycerol, was then added into the tubes. Oocytes were homogenized by passing through a 25-G needle ten times followed by 1 min of sonication. After rotating at 4 °C for 1 h and vortexed every 10 min for 5 s, samples were centrifuged at 13,000 rpm for 30 min at 4 °C. The supernatant was then collected and treated with 3× SDS loading buffer at 37 °C for 30 min. Cell lysate samples were run on 4–12% SDS-PAGE gels (Life Technologies) and blotted with the following antibodies. Primary (1:1000 dilution): mouse monoclonal anti-HA (BioLegend, 901503, clone 16B12), rabbit polyclonal anti-PKD2L1 (Millipore, AB9084) and mouse monoclonal anti-β-actin (GenScript, A00702). Secondary (1:10,000): IRDye 680-conjugated goat anti-mouse (LI-COR Biosciences, 926–68070) and IRD 800CW-conjugated goat anti-rabbit (LI-COR Biosciences, 926–32211). Images were scanned with the LI-COR Odyssey CLx imaging system.

### Electrophysiology

The cDNAs of the mouse PKD1L3 (NCBI accession number AY164486) encoding full-length and truncated CTD fragment (residues 1632–2151) of mPKD1L3 and the cDNAs of the full-length mouse PKD2L1 (NCBI accession number NM_181422.3) were cloned into a modified pGEMHE vector. The PKD1L3 constructs have an HA-tag fused to the N-terminus. The primers used in this study were list in Supplementary Table 2. cRNA was in vitro-synthesized and injected into Xenopus oocytes (30 ng/oocyte). The injected RNA has a molar ratio of PKD1L3: PKD2L1 = 2.5:1 to ensure an excess amount of PKD1L3 to eliminate the homomeric PKD2L1 complex formation. Injected oocytes were kept at 18 °C for 3–5 days and whole-oocyte currents were then recorded with a two-electrode voltage-clamp (TEVC) with an OC-725D Oocyte clamp amplifier (Warner Instruments), a Digidata 1440 A digitizer (Molecular Devices), and the pClamp 10 software (Molecular Devices). Standard bath solution contains 100 mM NaCl, 0.5 mM MgCl_2_, and 2 mM HEPES, pH 7.5. The pH 2.7 solution used for acid activation was generated by adding citric acid into the bath solution until pH reached 2.7. The Ca^2+^ solution used for Ca^2+^ activation was made by adding 10 mM CaCl_2_ into the bath solution.

A gap-free voltage protocol holding at −80 mV was used for TEVC recording. During recording, 10 mM Ca^2+^ was applied first for at least 70 s to test the Ca^2+^-induced activation followed by the Ca^2+^-induced inactivation. Then, after the Ca^2+^ was washed away, pH 2.7 solution was applied on the same oocyte for 15 s then washed away with the standard bath solution to trigger the acid-induced response. The sizes of the Ca^2+^-induced currents used in bar graphs were extracted at the peak of the current during 10 mM Ca^2+^ application, while that of the acid-induced current was measured at a time point of 20 s after the stop of the acid application/the start of the washing.

### Statistical analysis

Channel current comparators were done with GraphPad Prism, and the statistical significance was calculated with the unpaired two-sided Student’s *t* test. Results of *P* < 0.05 were considered as statistically significant (differences *P* < 0.05 are denoted by *, P < 0.01 by **, *P* < 0.001 by ***, and *P* < 0.0001 by ****). Results are presented as means ± SD.

### Reporting summary

Further information on research design is available in the Nature Research Reporting Summary linked to this article.

## Supplementary information

Supplementary Information

Reporting summary

## Data Availability

The atomic coordinates and EM maps for apo and Ca^2+^-loaded PKD1L3/PKD2L1 have been deposited in the PDB with the accession codes 7D7E and 7D7F, and the EMDB with the codes EMD-30606 and EMD-30607, respectively. Other data are available from the corresponding authors upon reasonable request. Source data are provided with this paper.

## Source data

Source Data
