# Supplementary material for: Structural basis for Ca2+ activation of the heteromeric PKD1L3/PKD2L1 channel
Source: Nat Commun. 2021 Aug 11;12:4871. doi: 10.1038/s41467-021-25216-z (PMC8357825; doi:10.1038/s41467-021-25216-z)
Supplement: Supplementary file 1 — Supplementary Information [file 41467_2021_25216_MOESM1_ESM.pdf]

Supplementary Figures and Legends

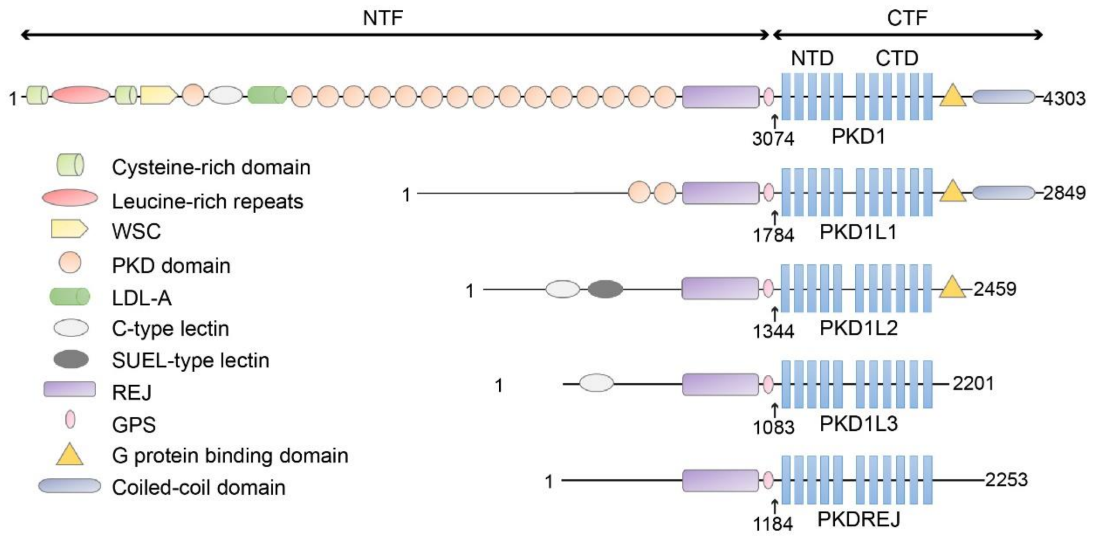

Supplementary Fig. 1 Diverse N-terminal fragment (NTF) and relatively conserved C-terminal fragment (CTF) of the PKD1 members. Shown here is the diagram of the domain organization of human PKD1 subfamily members. The 11-transmembrane helices are shown as light blue strips. Domain mapping follows the predication in UniProt (https://www.uniprot.org/). WSC: cell wall integrity and stress response component. LDL-A: low-density lipoprotein A. REJ: sperm receptor for egg jelly. GPS: G protein–coupled receptor proteolytic site. The Uniprot IDs of the aligned sequences are P98161(PKD1), Q8TDX9(PKD1L1), Q7Z442(PKD1L2), Q7Z443(PKD1L3), and Q9NTG1(PKDREJ).

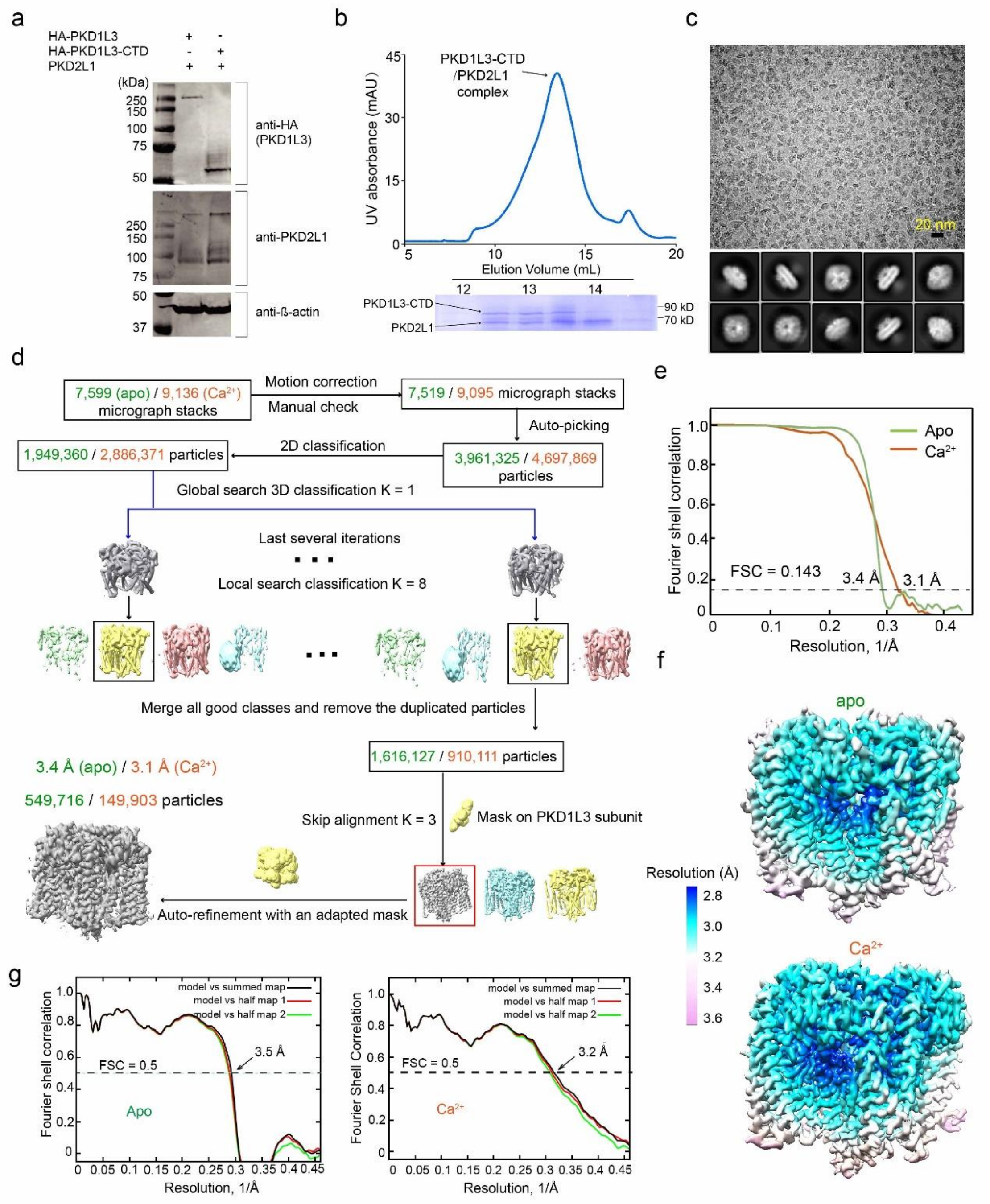

Supplementary Fig. 2 Structural determination of the PKD1L3-CTD/2L1 complex. a, Expression of the mouse full-length (FL) or truncated PKD1L3 (PKD1L3-CTD) with PKD2L1 in *Xenopus* oocytes for functional analysis. Shown here are representative western blots. The experiment was repeated three times independently with similar results. Rabbit polyclonal anti-PKD2L1 (Millipore), mouse monoclonal anti-HA (BioLegend), and mouse monoclonal anti-ß-actin (GenScript) were used as primary antibodies (1:1,000 dilution), and IRDye® 680RD goat anti-mouse and IRDye® 800CW goat anti-rabbit IgG (LI-COR

Biosciences) were used as secondary antibodies (1:10,000 dilution). **b**, Size exclusion chromatography purification of the mouse PKD1L3-CTD/PKD2L1 complex. The peak fractions were resolved by SDS-PAGE and visualized by Coomassie blue staining. The constructs used for structural analysis are mouse PKD1L3-CTD (residues 1632-2151) and PKD2L1 (residues 64-629). For simplicity, the truncated complex will be referred to as PKD1L3/2L1 hereafter. The experiment was repeated three times independently with similar results. **c**, Representative electron micrograph and 2D class averages. The experiment was repeated three times independently with similar results. **d**, Flowchart for EM data processing of apo (green) and Ca^{2+}-loaded (orange) heterocomplexes. Details can be found in Methods. **e**, Gold standard FSC curve of the structures for the 3D reconstruction. **f**, Local resolution maps for PKD1L3/2L1 complex in the absence (apo) or presence (Ca^{2+}) of 20 mM Ca^{2+}. The resolution heatmaps were calculated using RELION 3.0. **g**, FSC curves of the refined models versus the overall maps that it was refined against (black), of the model refined in the first of the two independent maps used for the gold-standard FSC versus that same map (red); and of the model refined in the first of the two independent maps versus the second independent map (green).

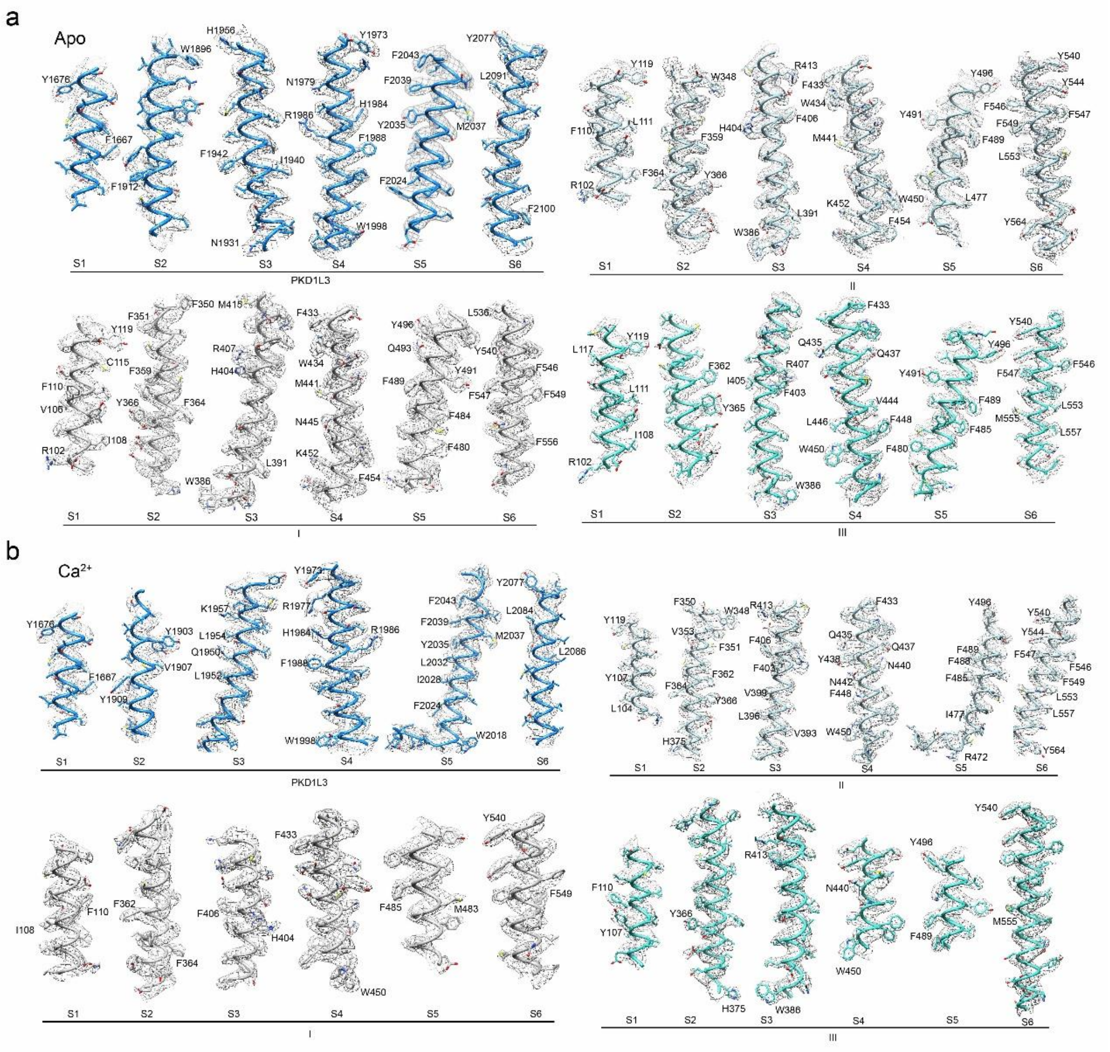

Supplementary Fig. 3 Representative EM maps for segments in the PKD1L3/2L1 complex. Densities for representative segments in the apo and Ca^{2+}-loaded complex are shown in **a** and **b**, respectively. The EM maps, contoured at 5 σ, were generated in UCSF Chimera. The representative bulky side chains used to facilitate sequence assignment are labeled.

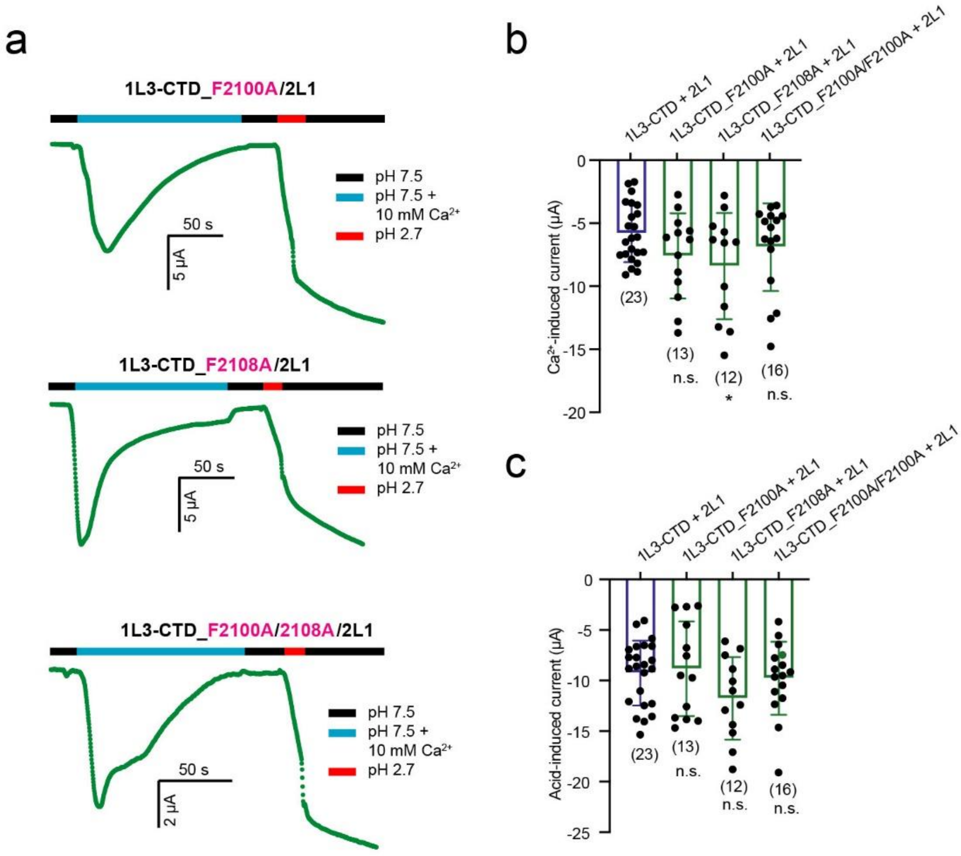

Supplementary Fig. 4 Gate mutations in PKD1L3 is not enough to open the gate. **a**, The mutant channels retains Ca^{2+} and acid activation. The Ca^{2+} and acid-induced currents were recorded from *Xenopus* oocytes expressing both PKD1L3-CTD with mutations and PKD2L1. Shown here are representative traces for gap-free recording at -80 mV. **b**, Scatter plots and bar graphs of the Ca^{2+}-induced currents at -80 mV recorded from oocytes expressing the indicated proteins. **c**, Scatter plots and bar graphs of the acid-induced currents at -80 mV recorded from oocytes expressing the indicated proteins. The number of oocytes is shown below each bar. Data are presented as mean ± S.D. in the bar graph. Currents in each group are compared with that of PKD1L3-FL/2L1-injected group with two-sided Student's t-test. n.s.: not significant; *P<0.05.

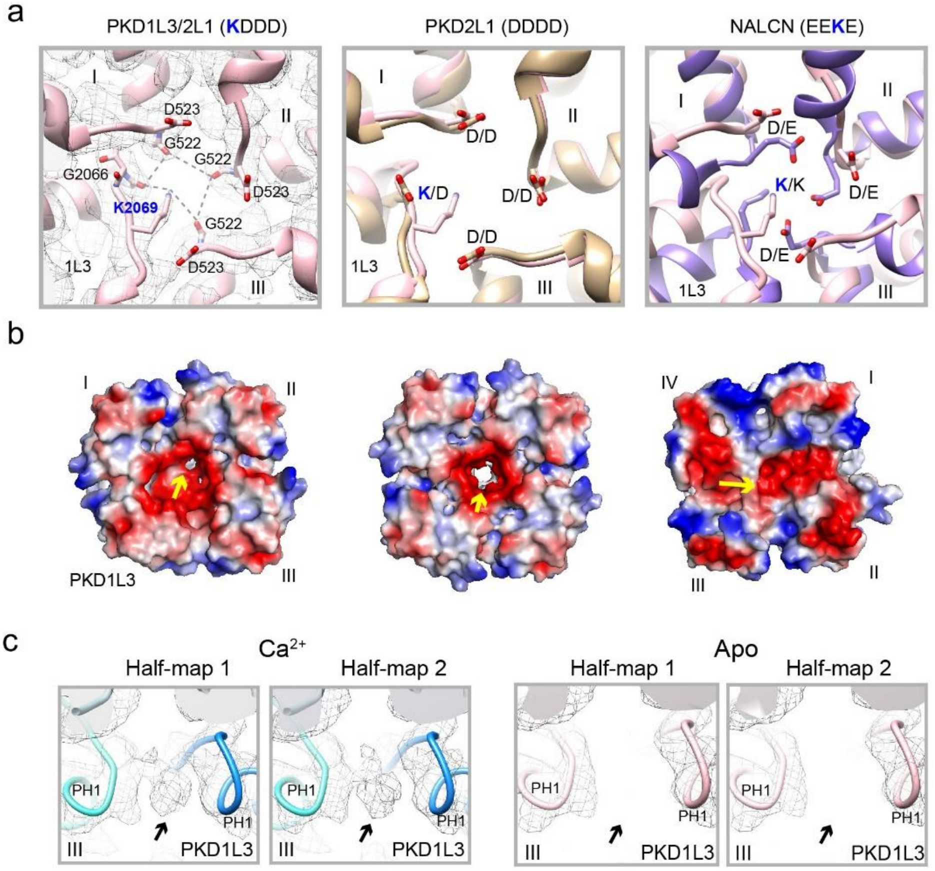

Supplementary Fig. 5 An asymmetric selectivity filter (SF). a, Structural comparison of the SF in different channels. *Left*: An intracellular view of the SF in the apo PKD1L3/2L1 complex. The densities, shown as gray mesh, are contoured at 5 σ. *Middle*: Superimposition of the SF from the PKD1L3/2L1 heterotetramer (pink) and the homo-tetrameric PKD2L1(gold, PDB: 5Z1W). The SF segments in the three PKD2L1 subunits remain the same as in the homotetramer. *Right*: Structural comparison with NALCN (purple, PDB: 7CM3), in which the SF is guarded by EEKE. **b**, Replacement of one PKD2L1 with PKD1L3 results in a narrower SF entrance that is off the central axis. Shown here are extracellular views of the electrostatic surface potential of the PD of PKD1L3/2L1 (left), homo-tetrameric PKD2L1 (middle), and NALCN (right). The entrance to the SF is indicated by the yellow arrow in each panel. The electrostatic surface potentials were calculated in PyMol. **c**, Extra density in the SF in the presence of added Ca^{2+} only. Shown here are half maps of the SF with (left, Ca^{2+}) or without (right, apo) 20 mM $CaCl_2$ that was added right before cryo-sample preparation. The maps are contoured at 5 σ.

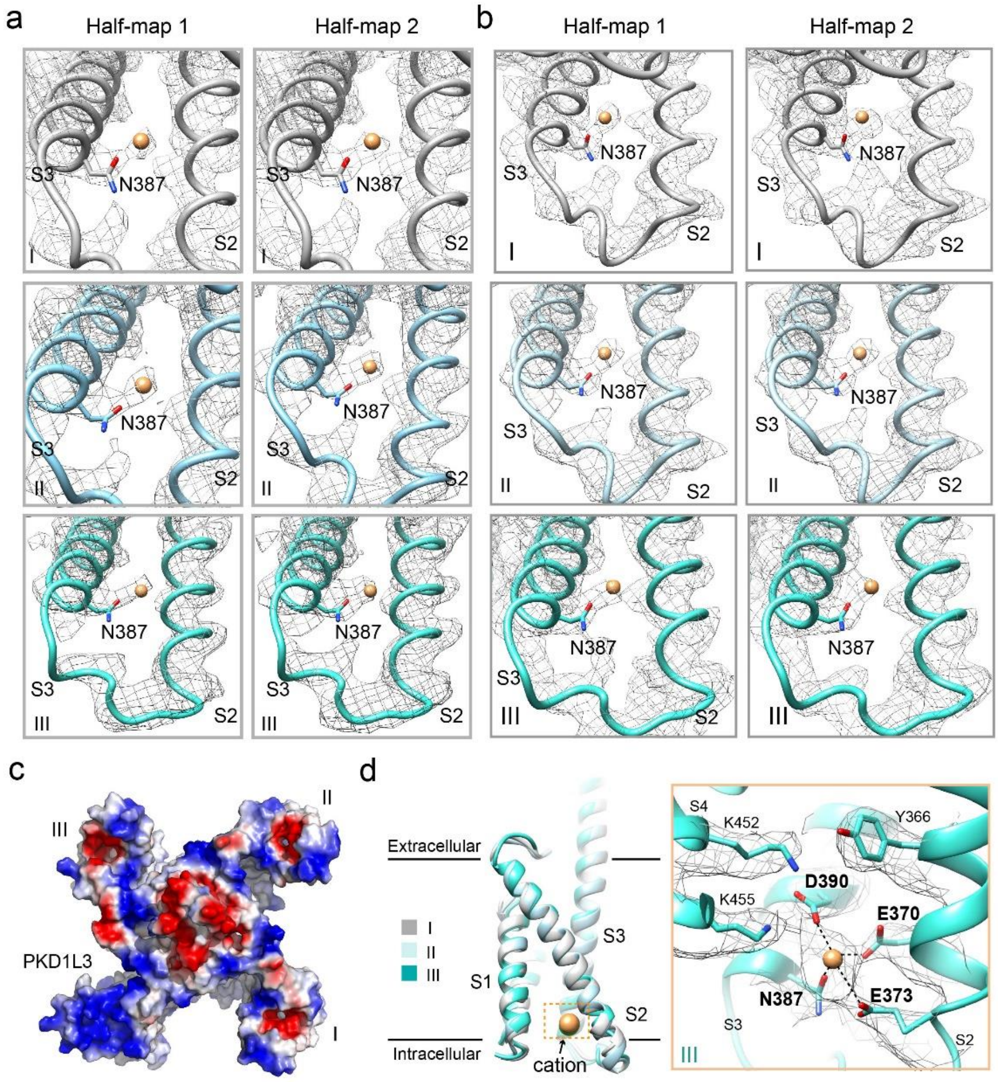

Supplementary Fig. 6 A cation binding site in the VSDs of the three PKD2L1 subunits, but not PKD1L3. a and **b**, In the maps for both apo and Ca^{2+}-loaded PKD1L3/2L1 complex, a similar extra density that may belong to a cation is contiguous with the side chain of Asn387 in both half-maps of the VSD of all three PKD2L1 subunits. The densities are shown as gray mesh and contoured at 5 σ in all panels. **c**, An acidic cytosol-facing cavity in each VSD of PKD2L1, but not PKD1L3. An intracellular view of the apo PKD1L3/2L1 complex is shown in surface electrostatic potential. PKD1L3-VSD is enriched of basic residues and lacks the cytosol-facing cavity. **d**, Identical cation binding site in the VSD of the three PKD2L1 subunits. *Inset*: The cation coordination site is accessible to the cytosol. The densities are contoured at 5 σ.

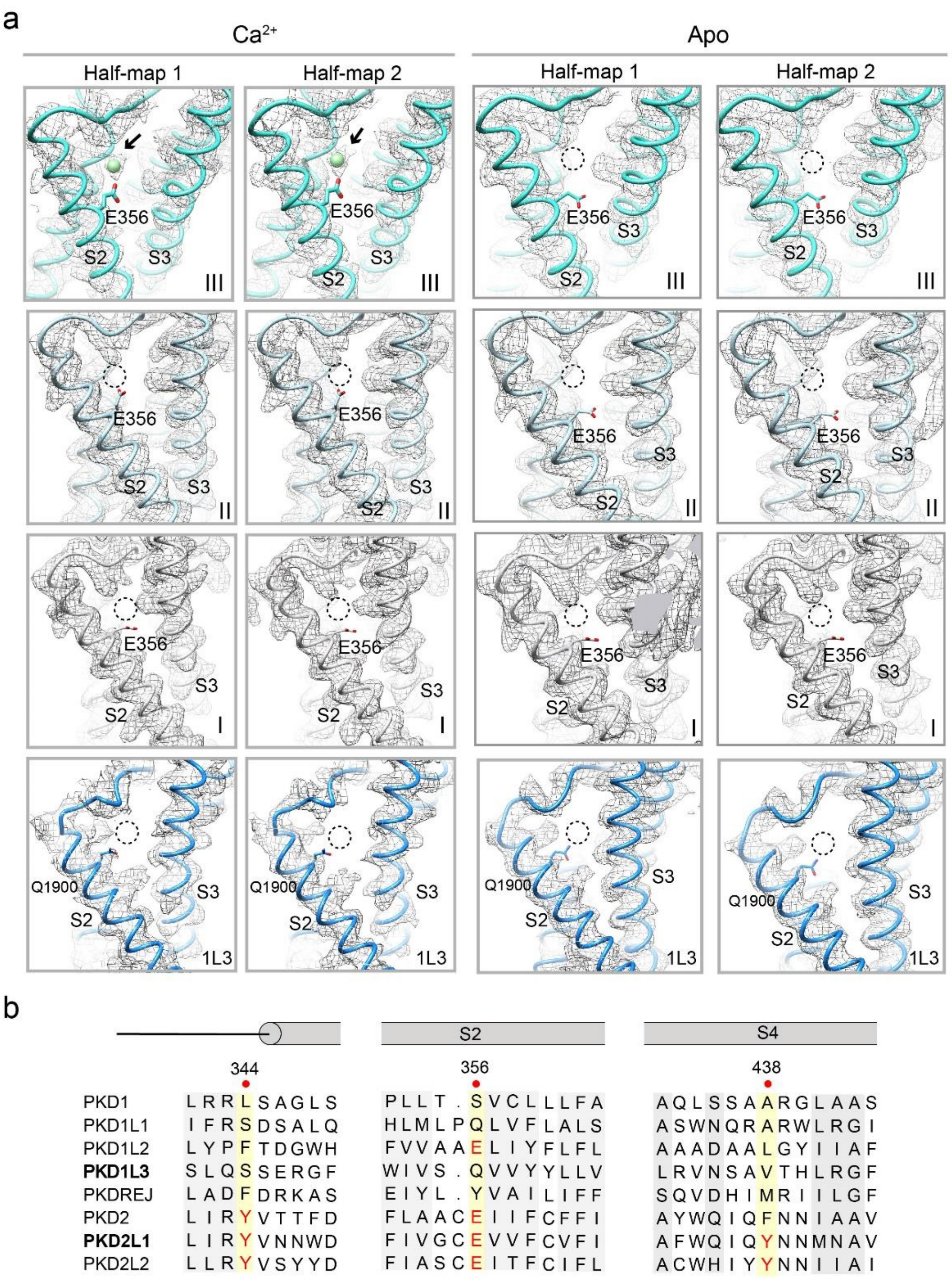

Supplementary Fig. 7 An unconventional Ca^{2+} binding site in VSD_{III} only. a, A spherical density is observed in the extracellular cleft of VSD_{III} in the Ca^{2+}-loaded complex only. Shown here are half maps of the four VSDs in Ca^{2+}-loaded (left) and apo (right) complex. The maps are contoured at 4 σ. **b**, The Ca^{2+} binding site is not conserved in PKD1 subfamily. Shown here is the sequence alignment of PKD1 and PKD2 subfamily members. The ion coordination residues are highlighted by red dots above.

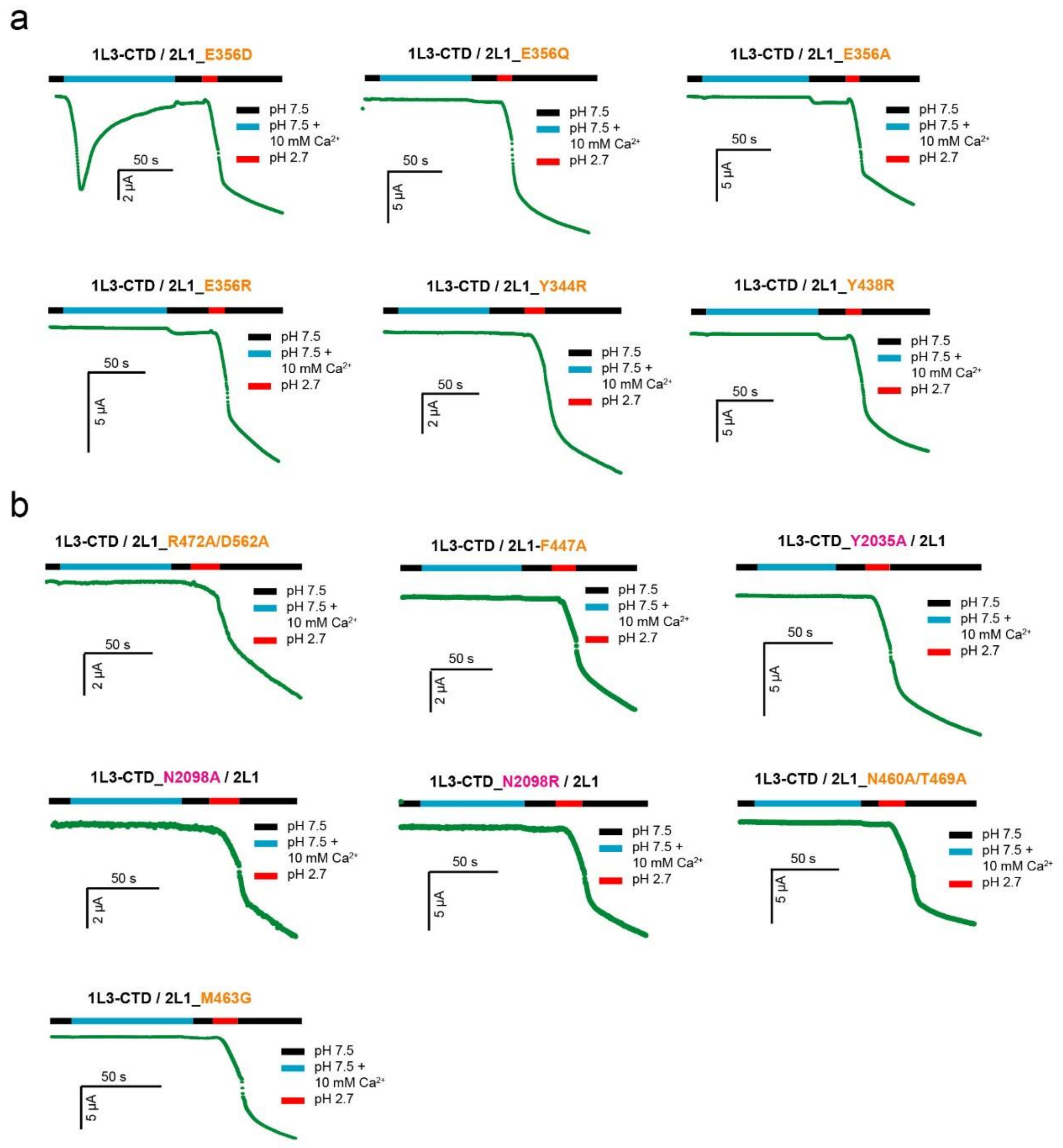

Supplementary Fig. 8 Representative traces of the PKD1K3 and PKD2L1 mutants that may alter Ca^{2+} activation. Representative traces for mutants in Fig. 4d and 4e are presented in **a** and **b**, respectively. Gap-free recording at -80 mV shows the Ca^{2+} and acid-induced currents from *Xenopus* oocytes expressing indicated proteins.

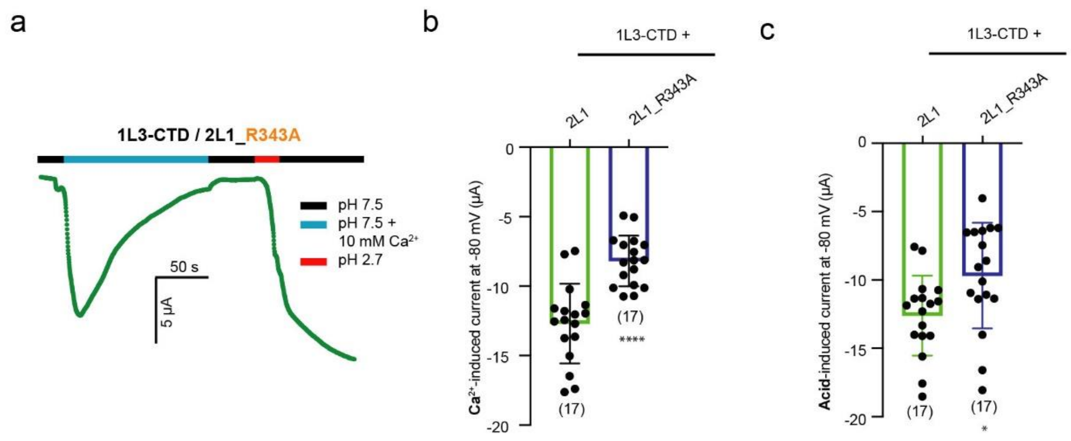

Supplementary Fig. 9 Electrophysiology of the mutation of R343A in PKD2L1. a, Representative traces for mutants R343A in PKD2L1. Gap-free recording at -80 mV shows the Ca^{2+} and acid-induced currents from *Xenopus* oocytes expressing indicated proteins. Bar graphs of Ca^{2+} and acid-induced currents by mutants are shown in **b** and **c**, respectively. Data are presented as mean ± S.D. in the bar graph. Currents of other groups are compared with that injected with PKD1L3-CTD/PKD2L1 with two-sided Student's t-test. *P<0.05; ****P<0.0001.

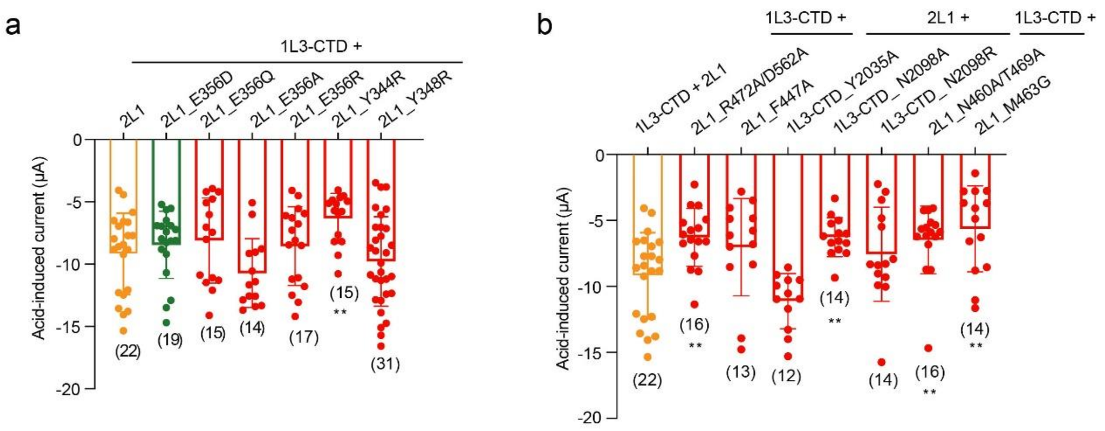

Supplementary Fig. 10 PKD1L3/2L1 mutations that abolish Ca^{2+}-induced current have little or no effect on acid-induced channel activity. Scatter plots and bar graphs of the acid-induced currents by mutants shown in Fig. 4d and 4e are presented in panels **a** and **b**, respectively. Bar graphs of acid-induced currents by mutants that abolish Ca^{2+}-induced current are colored red, while those of WT and PKD1L3/2L1-E356D that preserves Ca^{2+}-induced current are colored yellow and green, respectively. Data are presented as mean ± S.D. in the bar graph. No significant or mild changes were found when compared the currents of mutants with that of PKD1L3-CTD/PKD2L1 with two-sided Student's t-test.

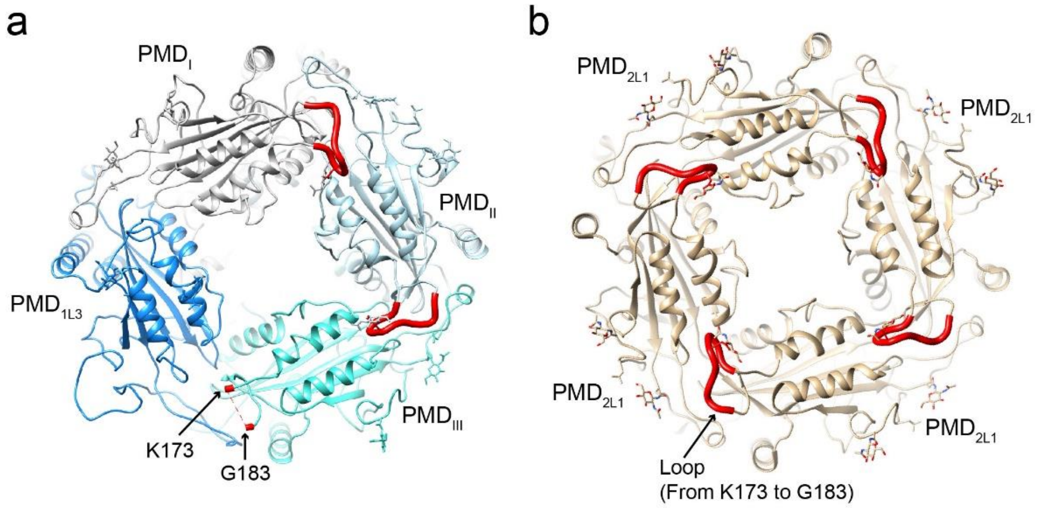

Supplementary Fig. 11 Different assembly manner of PMDs of PKD1L3/2L1 complex and PKD2L1. Top view of PKD1L3/2L1 complex and homotetrameric PKD2L1 are presented in panels **a** and **b**, respectively. The loops mediated PMD assembly are highlighted by red color.

Supplementary Table 1 Statistics for data collection and model refinement

	PKD1L3/PKD2L1	PKD1L3/PKD2L1 + 20 mM Ca^{2+}
**Data collection**		
EM equipment	FEI Titan Krios	FEI Titan Krios
Magnification	×81000	×81000
Voltage (kV)	300	300
Detector	K3	K3
Pixel size (Å)	1.087	1.087
Electron dose (e-/Å^2)	50	50
Defocus range (μm)	-1.0~-2.3	-1.0~-2.3
Micrographs	7599	9136
**Reconstruction**		
Software	RELION 3.0	RELION 3.0
EMDB code	30606	30607
Number of particles	549,716	228,940
Symmetry	C1	C1
Final resolution (Å)	3.4	3.1
Map sharpening B-factor (Å^2)	-84.86	-75.0
**Model building**		
Software	Coot 0.8.2	Coot 0.8.2
Refinement	Phenix	Phenix
PDB code	7D7E	7D7F
**Validation**		
r.m.s deviations		
Bonds length (Å)	0.007	0.01
Bonds Angle (°)	0.652	0.773
Ramachandran plot		
Preferred (%)	90.85	92.16
Allowed (%)	9.10	7.68
Outlier (%)	0.05	0.16
MolProbity score	2.54	2.52

Supplementary Table 2 The primers for molecular cloning

Name	Sequence
F-pCAG-1L3-NotI	AAGGAAAAAGCGGCCGCCCCCATCTACACTGCCCCAGC
R-pCAG-1L3-XhoI	CCGCTCGAGTCAACCAGTGTTGTCAGCGTGTTCC
F-pCAG-2L1-NotI	AAGGAAAAAGCGGCCGCCACCCTGGTGTCCAGCTGC
R-pCAG-2L1-XhoI	CCGCTCGAGTCACCCCAGTTCCCTCAAGGTGC
1L3-CTD-Q5-F	AGATTATGCTGGAGGTCCCATCTACACTGCCCCA
1L3-CTD-Q5-R	GGAACATCATATGGATACATGGTGGATGGGGAATTG
1L3-CTD-Y2035A-Q5-F	GTTAAGCAGCGCCGCCATGACTTTCAAC
1L3-CTD-Y2035A-Q5-R	AGGACCACGATGATCAGT
1L3-CTD-N2098A-Q5-F	TGTGATCATTGCCCTTTTTGTTTCTGC
1L3-CTD-N2098A-Q5-R	AGTCCCATCAAGATGATG
1L3-CTD-N2098R-Q5-F	TGTGATCATTCGCCTTTTTGTTTCTGC
1L3-CTD-N2098R-Q5-R	AGTCCCATCAAGATGATG
2L1-R343A-Q5-F	TAAGCTGATCGCCTATGTGAATAACTGGGACTTC
2L1-R343A-Q5-R	ACTGTGCGGATCTGCCAG
2L1-Y344R-Q5-F	GCTGATCCGCCGTGTGAATAACTGG
2L1-Y344R-Q5-R	TTAACTGTGCGGATCTGC
2L1-E356A-Q5-F	TGTGGGCTGTGCAGTTGTCTTCT
2L1-E356A-Q5-R	ATGAAGAAGTCCCAGTTATTCAC
2L1-E356R-Q5-F	TGTGGGCTGTCGAGTTGTCTTCT
2L1-E356R-Q5-R	ATGAAGAAGTCCCAGTTATTC
2L1-E356Q-Q5-F	TGTGGGCTGTCAAGTTGTCTTCT
2L1-E356Q-Q5-R	ATGAAGAAGTCCCAGTTATTCAC
2L1-E356D-Q5-F	TGTGGGCTGTGACGTTGTCTTCT
2L1-E356D-Q5-R	ATGAAGAAGTCCCAGTTATTCAC
2L1-Y438R-Q5-F	GCAGACTCAGCGCAATAACATGAACGC
2L1-Y438R-Q5-R	CAGAAGGCCAGGAACTCA
2L1-F447A-Q5-F	GGTCAACCTTGCCTTTGCTTGGATC
2L1-F447A-Q5-R	GCGTTCATGTTATTGTACTG
2L1-N460A/T469A-Q5-F	CAGCTCTCCTCCGCCCTGGCTCGCTGTGCCAAG
2L1-N460A/T469A-Q5-R	TGTCATGGTCTTGGCGAAGCTGATATACTTGAATATCTTGATC CAAG
PKD2L1-M463G-Q5-F	CAACAAGACCGGAACACAGCTCTC
PKD2L1-M463G-Q5-R	AAGCTGATATACTTGAATATCTTG
2L1-R472A-Q5-F	CACCCTGGCTGCCTGTGCCAAGG
2L1-R472A-Q5-R	GAGGAGAGCTGTGTCATG

2L1-D562A-Q5-F	CATCATCAACGCCACATACTCCG
2L1-D562A-Q5-R	GCCAGGAACATGTTCAGG
