# Supplementary material for: Structural basis for Ca2+ activation of the heteromeric PKD1L3/PKD2L1 channel
Source: Nat Commun. 2021 Aug 11;12:4871. doi: 10.1038/s41467-021-25216-z (PMC8357825; doi:10.1038/s41467-021-25216-z)
Supplement: Supplementary file 2 — Reporting summary [file 41467_2021_25216_MOESM2_ESM.pdf]

nature portfolio

Corresponding author(s): Qiang Su, Yong Yu, Yigong Shi

Last updated by author(s): Jul 15, 2021

Reporting Summary

Nature Portfolio wishes to improve the reproducibility of the work that we publish. This form provides structure for consistency and transparency in reporting. For further information on Nature Portfolio policies, see our Editorial Policies and the Editorial Policy Checklist.

Statistics

For all statistical analyses, confirm that the following items are present in the figure legend, table legend, main text, or Methods section.

n/a	Confirmed	
☐	☒	The exact sample size (n) for each experimental group/condition, given as a discrete number and unit of measurement
☐	☒	A statement on whether measurements were taken from distinct samples or whether the same sample was measured repeatedly
☐	☒	The statistical test(s) used AND whether they are one- or two-sided<br>*Only common tests should be described solely by name; describe more complex techniques in the Methods section.*
☒	☐	A description of all covariates tested
☒	☐	A description of any assumptions or corrections, such as tests of normality and adjustment for multiple comparisons
☐	☒	A full description of the statistical parameters including central tendency (e.g. means) or other basic estimates (e.g. regression coefficient) AND variation (e.g. standard deviation) or associated estimates of uncertainty (e.g. confidence intervals)
☐	☒	For null hypothesis testing, the test statistic (e.g. F, t, r) with confidence intervals, effect sizes, degrees of freedom and P value noted<br>*Give P values as exact values whenever suitable.*
☒	☐	For Bayesian analysis, information on the choice of priors and Markov chain Monte Carlo settings
☒	☐	For hierarchical and complex designs, identification of the appropriate level for tests and full reporting of outcomes
☒	☐	Estimates of effect sizes (e.g. Cohen's d, Pearson's r), indicating how they were calculated

Our web collection on statistics for biologists contains articles on many of the points above.

Software and code

Policy information about availability of computer code

Data collection	Titan Krios electron microscope (Thermo Fisher) operating at 300 kV and equipped with Gatan K2 Summit detector, and GIF Quantum energy filter.
Data analysis	The data was processed and analyzed by RELION 3.1, MotionCor2, UCSF Chimera, WinCoot 0.8.9 and Phenix 1.17.

For manuscripts utilizing custom algorithms or software that are central to the research but not yet described in published literature, software must be made available to editors and reviewers. We strongly encourage code deposition in a community repository (e.g. GitHub). See the Nature Portfolio guidelines for submitting code & software for further information.

Data

Policy information about availability of data

All manuscripts must include a data availability statement. This statement should provide the following information, where applicable:

- Accession codes, unique identifiers, or web links for publicly available datasets
- A description of any restrictions on data availability
- For clinical datasets or third party data, please ensure that the statement adheres to our policy

The atomic coordinates and EM maps for apo and Ca2+-loaded PKD1L3/PKD2L1 have been deposited in the PDB with the accession codes 7D7E and 7D7F, and the EMDB with the codes EMD-30606 and EMD-30607, respectively.
The Uniprot IDs of the aligned sequences used in this study are P98161(PKD1), Q8TDX9(PKD1L1), Q7Z442(PKD1L2), Q7Z443(PKD1L3), and Q9NTG1(PKDREJ).The Uniprot IDs of the aligned sequences are P98161(PKD1), Q8TDX9(PKD1L1), Q7Z442(PKD1L2), Q7Z443(PKD1L3), and Q9NTG1(PKDREJ).

Field-specific reporting

Please select the one below that is the best fit for your research. If you are not sure, read the appropriate sections before making your selection.

- [x] Life sciences
- [ ] Behavioural & social sciences
- [ ] Ecological, evolutionary & environmental sciences

For a reference copy of the document with all sections, see nature.com/documents/nr-reporting-summary-flat.pdf

Life sciences study design

All studies must disclose on these points even when the disclosure is negative.

Sample size	In our electrophysiology recording, more than 12 oocytes were tested for each mutants. The number is selected based on our previous experience and what people generally do in electrophysiology filed. This size is sufficient for performing the statistical test. Sample size was not applied to the structural analysis.
Data exclusions	No data was excluded in structural analysis. Electrophysiology recording data from a small number of oocytes that have big leak currents (due to bad oocyte quality) were excluded.
Replication	Replication was not applied to the structural analysis. All recording data have been successfully repeated with at least two to three batches of oocytes and all results were similar. Western blot is also repeated for three times successfully.
Randomization	Randomization was not applied to the structural analysis. Xenopus oocytes were randomly selected for injection and current recording.
Blinding	Blinding was not applied to structural analysis. Blinding was not performed in electrophysiology recording since the protocol was straightforwad and was performed identically to the WT and mutants by computer controlled equipment. Data analysis was also performed by computer based method.

Reporting for specific materials, systems and methods

We require information from authors about some types of materials, experimental systems and methods used in many studies. Here, indicate whether each material, system or method listed is relevant to your study. If you are not sure if a list item applies to your research, read the appropriate section before selecting a response.

Materials & experimental systems

n/a	Involved in the study	
[ ]	[x]	Antibodies
[ ]	[x]	Eukaryotic cell lines
[x]	[ ]	Palaeontology and archaeology
[x]	[ ]	Animals and other organisms
[x]	[ ]	Human research participants
[x]	[ ]	Clinical data
[x]	[ ]	Dual use research of concern

Methods

n/a	Involved in the study	
[x]	[ ]	ChIP-seq
[x]	[ ]	Flow cytometry
[x]	[ ]	MRI-based neuroimaging

Antibodies

Antibodies used	Primary antibodies: Rabbit polyclonal anti-PKD2L1 (Millipore, AB9084), mouse monoclonal anti-HA (BioLegend, 901503, clone 16B12), and mouse monoclonal anti-ß-actin (GenScript, A00702). Secondary antibodies: IRDye® 680RD goat anti-mouse (LI-COR Biosciences, 926-68070) and IRDye® 800CW goat anti-rabbit IgG (LI-COR Biosciences,, 926-32211).
Validation	All antibodies are validated with negative control samples in our lab. The mouse anti-HA antibody from BioLegend and mouse anti-ß-actin antibody from GenScript has been widely used in literature including previous publication from us.

Eukaryotic cell lines

Policy information about cell lines

Cell line source(s)	The cell lines of HEK293F were purchased from ATCC.
Authentication	The cell lines were not authenticated.
Mycoplasma contamination	The cell lines were not tested for mycoplasma contamination.
Commonly misidentified lines (See ICLAC register)	N.S.
